# Neonatal resuscitation after birth: Swedish midwives’ experiences of and perceptions about separation of mothers and their newborn babies

**DOI:** 10.18332/ejm/162319

**Published:** 2023-05-18

**Authors:** Pyrola Bäcke, Li Thies-Lagergren, Ylva Thernström Blomqvist

**Affiliations:** 1 Neonatal Intensive Care Unit, University Hospital, Uppsala, Sweden; 2 Department of Women’s and Children’s Health, Uppsala University, Uppsala, Sweden; 3 Midwifery Research – Reproductive, Perinatal and Sexual Health, Department of Health Sciences, Faculty of Medicine, Lund University, Lund, Sweden

**Keywords:** midwifery, experiences, neonatal resuscitation, emergency care, skin-to-skin care, zero separation

## Abstract

**INTRODUCTION:**

This study aimed to investigate midwives’ experiences of and perceptions about mother–baby separation during resuscitation of the baby following birth.

**METHODS:**

A qualitative study was conducted using an author-designed questionnaire. Fifty-four midwives from two Swedish birth units with different working methods regarding neonatal resuscitation – at the mother’s bedside in the birth room or in a designated resuscitation room outside the birth room – completed the questionnaire. Data were analyzed using qualitative content analysis.

**RESULTS:**

Most midwives had experience of removing a newborn baby in need of critical care from the birth room, thus separating the mother and baby. The midwives identified the difficulties and challenges involved in carrying out emergency care in the birth room after birth and had divergent opinions about what they considered possible in these birth situations. They agreed on the benefits, for both mother and baby, in performing emergency care in the birth room and avoiding a separation altogether, if possible.

**CONCLUSIONS:**

There are good opportunities to reduce separation of mother and baby after birth; training, knowledge, education and the right environmental conditions are important factors in successfully implementing new ways of working. It is possible to work towards reducing separation and this work should continue and strive to eliminate separation as far as possible.

## INTRODUCTION

The practice of skin-to-skin care (SSC) immediately after the birth of a healthy newborn baby is today standard care in many countries worldwide and it is well known that SSC promotes a more stable heart activity and enhances oxygen saturation in the newborn, as well as leading to less crying^1^. Skin-to-skin care also promotes initiation of breastfeeding and babies tend to breastfeed for longer when on SSC immediately after birth^1,2^. Furthermore, SSC enhances mother–baby bonding^2^ and the physiological and psychological benefits of SSC have health advantages for the baby and the mother, in the short-term and long-term^3^. The World Health Organization (WHO) as well as the United Nations International Children’s Emergency Fund (UNICEF) recommend providing SSC immediately after birth^4,5^.

Separation of the mother–baby dyad should be performed only when medically indicated^4^. Despite this, Tasseau et al.^6^ found that many healthy newborn babies are separated from their mothers during the first two hours after birth, mainly for routine care. Separation can be caused by maternal factors where emergency care is needed, such as major postpartum hemorrhage or when the mother needs a surgical procedure in an operating theatre. Separation can also be due to factors necessitating neonatal resuscitation. The psychological effects of separation of mother and baby immediately after birth may include experience of being deprived of the first hours together and may disturb and postpone bonding^7^.

Neonatal resuscitation involves emergency care of a seriously ill/preterm baby after birth. Recommendations are available to guide caregivers on the structure of resuscitation actions^8,9^. When needed, neonatal resuscitation following birth can be performed on a neonatal resuscitation table, either at the mother’s bedside (termed ‘motherside resuscitation’) in the birth room, or in a room next to the birth room designed for neonatal resuscitation. Motherside resuscitation has been found to be valued by parents; it allows parents to see and touch their baby, and to observe what the clinical team is doing^10^. Further, it enhances an opportunity for early contact and involvement as it allows the parents to share the first moments of their newborn baby’s life^11^. Also, motherside neonatal resuscitation care allows the other parent to be both with the baby and the mother. In general, parents who have had the experience are positive to motherside neonatal resuscitation and do not regret having been present, despite strong emotions while watching their baby being resuscitated^11^. To avoid separation between mothers and babies after birth requires system changes including planning and organization of care, equipment, and design of units^12^.

A qualitative interview study reported that clinicians had some concerns regarding motherside resuscitation related to being observed closely by the parents. It described, however, that most of the interviewed clinicians were positive to motherside resuscitation^10^.

Regarding midwives’ experiences of and perceptions about baby–mother separation during resuscitation of newborns, one survey study reported that midwives in a Canadian hospital setting were reluctant to carry out motherside resuscitation because of practical and organizational obstacles^13^. However, considering the positive effects of non-separation of the newborn baby and the mother, it is important to further investigate midwives’ attitudes to neonatal resuscitation in different settings. Therefore, the aim of the present study was to investigate midwives’ experiences of and perceptions about baby–mother separation during resuscitation of the baby following birth.

## METHODS

This survey combined qualitative and quantitative data^14^ to gain an increased understanding and knowledge regarding midwives’ experiences of and perceptions about mother–baby separation when the baby needs to be resuscitated after birth.

### Setting and sample

This study was conducted at two Swedish birth units (herein referred to as unit A and unit B) that are organized in different ways regarding neonatal resuscitation. At the time of the study, there were 46 birth units in Sweden and anecdotally practice differs among them regarding initiating neonatal resuscitation at the mother’s bedside on a designated table or while on SSC in the birth room, or in a designated resuscitation room outside the birth room. Further, no data on place of resuscitation in these units is available.

At birth unit A, the newborn baby’s health is assessed by the midwife and evaluated for signs of respiratory disorder. If resuscitation is needed, SSC is stopped, and the baby is taken to a separate room with a radiant warmer where evaluations and resuscitation can be performed. The midwife always puts on an identity band on the baby before cutting the umbilical cord. At birth unit B, midwives, neonatal nurses, and neonatologists have for more than two decades performed neonatal evaluations and resuscitation in the birth room without separating the baby from the mother (motherside resuscitation). Some measurements, such as oxygen flow rate and oxygen saturation, are performed while on SSC on the mother’s breast, if possible, otherwise on a radiant warmer at the mother’s bedside, inside the birth room.

Inclusion criteria were all midwives (n=81) working in birth units A and B; exclusion criteria were others working at the units, such as physicians and assistant nurses.

### Data collection

The questionnaire was designed by the authors, based on their clinical experience and knowledge from current research^1,2,10,13^. To ensure content validity, the questions were pilot tested on four registered nurses and midwives working at a neonatal intensive care unit, but with experience of baby resuscitation both inside and outside the birth room. Thereafter, small adjustments were made. The questionnaires were in Swedish and contained a total of 16 items; six questions with fixed answer alternatives and ten open-ended questions. The questions with fixed answers concerned the midwives’ age and number of years in the profession, and questions such as whether they had ever participated in the resuscitation of a newborn baby, either on a resuscitation table in the birth room or SSC (yes/no question). The open-ended questions related to the midwives’ own experiences of situations such as taking a newborn baby out of the birth room to an emergency room or giving a baby initial assistance to support breathing immediately after birth. The open-ended questions also asked how the midwives felt about initial respiratory support for a newborn baby while on SSC or on a resuscitation table elsewhere in the birth room, as well as about the midwives’ opinion of advantages and disadvantages of referring a newborn baby to an emergency room versus neonatal resuscitation while on SSC or elsewhere in the birth room.

All midwives who worked in the two birth units received oral and written information about the study from the head midwife, including an invitation to participate by answering the questionnaire (either on paper or as a web survey).

### Ethics considerations

This study was performed in accordance with the ethical standards of the Helsinki Declaration^15^. Permission to perform the study was obtained from the medical directors and head midwife of the two birth units. The midwives received written information about the aim of the study, including the assurance that their participation would be voluntary and that they would have the right to withdraw at any time without giving any explanation. Anonymity was assured as the questionnaires were uncoded and the participants answered anonymously.

### Statistical analysis

Qualitative data collected were analyzed using content analysis, as described by Lindgren et al.^16^. All answers to the open-ended questions were analyzed together and the analysis focused on finding similarities and differences in the text related to the study’s aim. As a first step in the analysis, meaning units were identified in the text; thereafter, the meaning units were given codes serving as titles for separate meaning units describing their contents. Categories were established by searching for similarities and differences in the codes and the text. The entire analysis took place in Swedish before everything was translated into English in connection with the manuscript writing. During the analysis process, the authors together reflected on and discussed the interpretation of the data.

The quantitative data were analyzed with descriptive statistics, using SPSS (version 22; SPSS Inc., Chicago, IL) software.

## RESULTS

A total of 54 (67%) midwives completed the questionnaire, 36 (67%) from birth unit A and 18 (33%) from birth unit B. Their ages ranged 28–68 years (mean: 44), and they had worked as midwives for 1–42 years (mean: 13).

The qualitative content analysis resulted in four categories (Table 1) regarding midwives’ experiences of and perceptions about baby–mother separation during the baby’s resuscitation after birth.

**Table 1:** Overview of categories

*Categories*
Take a newborn baby out of the birth room
Work towards reducing separation
Difficulties and challenges of providing emergency care for the baby in the birth room
Benefits of reduced separation

### Take a newborn baby out of the birth room

In all, 45 (83%) of the midwives stated that they had on some occasion separated mother and baby and taken the baby to a resuscitation room (Table 2). There were differences between the birth units; all midwives in unit B answered that they had been involved in the resuscitation of a newborn baby in the birth room, while only 3 (8%) of the midwives in unit A had the same experience. Whereas all midwives in unit A had been involved in taking a newborn baby from the birth room to a resuscitation room, 9 (50%) from unit B had the same experience.

**Table 2:** Midwives’ experiences of resuscitating a newborn baby

*Questions*	*All (N=54) n (%)*	*Birth unit A (N=36) n (%)*	*Birth unit B (N=18) n (%)*
Have you ever participated in taking a newborn baby out of the birth room to a resuscitation room? (yes)	45 (83)	36 (100)	9 (50)
Have you been involved in the resuscitation of a newborn baby in the birth room? (yes)	21 (39)	3 (8)	18 (100)
Have you been involved in carrying out resuscitation of a newborn baby lying skin-to-skin on the mother’s breast? (yes)	7 (13)	2 (6)	5 (28)

According to responses to the open-ended questions, the reasons why the midwives chose to take a newborn baby out of the birth room included low Apgar score, the baby’s need for respiratory support, and critical illness of the baby, necessitating advanced care with help from the neonatal intensive care unit. Some midwives said that one reason for taking a baby to the resuscitation room was that the baby was expected to be in a bad condition, for instance when the baby was born preterm. One participant also mentioned that pediatricians sometimes request that a baby be taken to the emergency room for a specific reason.

The midwives from birth unit B stated that they do not take newborn babies out of the birth room because the radiant warmer is located inside the birth room. They recalled a few isolated cases where a baby did need to be taken out of the birth room and separated from its mother and said that the most likely reason was that it was known before the birth that the baby would need neonatal care. Overall, the midwives expressed that in cases where there has been a normal pregnancy and birth, and where the mother is healthy and the baby is expected to be healthy but is tired after birth, they choose to wait and see, before taking the baby to a resuscitation table. They also described that they generally choose to wait for a baby to have normal tonus or pulse, and for cutaneous stimulation to take effect. One midwife stated that they also choose to wait and see when a baby is born preterm; she added that in such cases, the placental transfusion is extra important:

*‘Begin with cutaneous stimulation first to see if the baby is recovering within a reasonable time. If it does not go in the right direction, I cut the umbilical cord and take the baby out of the birth room, but I always try to give the baby a chance first.’*

### Work towards reducing separation

Of the midwives, 21 (39%) had, at some stage, performed neonatal resuscitation in the birth room, and 7 (13%) had carried out resuscitation on SSC with the mother (Table 2). Based on their descriptions, procedures that could be performed with the baby on SSC included respiratory support such as continuous positive airway pressure, saturation measurement, and cleaning the baby’s airways, as well as cutaneous stimulation for the baby to ‘get started’. Several midwives felt that all resuscitation procedures on a newborn baby can be performed inside the birth room, as well as more advanced care such as intubation and navel catheterization. However, some of the midwives in unit A believed that advanced resuscitation cannot be performed in the birth room as there may be difficulties in performing it on SSC with the mother; also, they said that the furniture in the birth room is not adapted for this type of care:

*‘… If the conditions are met, I think that resuscitation can be done with the parents in the birth room.’*

In unit B, the routine for a long time has been that newborn resuscitation is performed in the birth room provided there is sufficient space. Answering the question about resuscitation of the baby during SSC with the mother, the midwives in unit B identified which procedures can be performed with the baby on SSC on the mother’s chest and which need to be done on the resuscitation table in the birth room. They described that initial respiratory support often occurs on the mother’s chest, and that even more advanced care, such as cardiac compressions, can be performed on the mother’s chest while on SSC. Intubation and umbilical catheterization were considered by many to be too complicated to perform while on SSC; these procedures, they said, should rather be performed at the mother’s bedside, but still in the birth room.

### Difficulties and challenges of providing emergency care for the baby in the birth room

According to the midwives, severe complications such as retained placenta took precedence over providing immediate care for the baby when on SSC with the mother. Sometimes, on the other hand, the mother’s need for care may be forgotten if the focus is on the care of the baby. In such situations, it may be difficult for a midwife to simultaneously care for both the baby and the mother, and to prioritize the care:

*‘If the mother herself needs urgent help, for example if she is bleeding a lot or if the placenta is sitting and you need to press the mother's stomach [uterus] while at the same time ventilating the baby lying there, this can delay the mother's care and make it difficult.’*

One thing the midwives highlighted was a lack of space in the birth room when a lot of staff were present, which could mean difficulties in getting a good working position and at the same time ensuring a good position of the baby to achieve effective ventilation. Lack of space was also perceived to pose a medical risk to the mother if in need of urgent care. Some suggested transferring the baby to a resuscitation table in the birth room in the event of the mother having an acute complication.

Some midwives described how a mother can feel exposed and uncovered in connection with birth. During resuscitation of the baby, there can be many people inside the birth room, which could make the mother feel uncomfortable. Some midwives also expressed the importance of being attentive to the mother’s/parents’ wishes regarding whether the resuscitation of the baby should take place in another room instead of inside the birth room:

*‘… She can feel naked and vulnerable with many people around her. It is important to think about the [mother's] integrity and that she may need to “land” for a little while. Many women need that.’*

Resuscitation of the baby can be traumatic and scary for parents. The midwives described how the situation can be perceived as chaotic, where stressed staff are unable to provide support to the mother. They realized that a mother may experience fear that her baby will not survive and that she may feel inadequate as a mother. They concluded that resuscitation at the mother’s bedside may not be suitable for all mothers and families:

*‘Although I think many parents experience it as positive, I think it will stress others. I think it can lead to parents feeling that they are not enough, like they should be doing something …’*

In a resuscitation situation, parents who become worried and scared can distract staff from their focus on the baby and prevent them from working undisturbed. Many of the midwives suggested that certain staff should be dedicated to supporting and informing parents during the resuscitation process:

*‘Sometimes it is easier to work only on the baby without needing to inform the parents [about the procedure]. Then you can focus 100% on the baby.’*

Several of the midwives proposed that greater emphasis should be placed on midwives’ education and practical training to enable optimal care of the baby. The training of neonatal staff should also be intensified. Performing SSC breathing support on the mother’s breast, for instance, requires that the baby be held in a good position to optimize ventilation of the lungs. If this is not carried out properly, there may be a delay in the effect. The clinical service column in the birth room is located next to the birth bed, which, according to the midwives, enables quick interventions that would be delayed if the baby were transferred to an emergency room:

*‘The use of the equipment because “it is there” makes an emergency easier to deal with than if you had to wait for a minute to …’*

Several additional challenges of providing emergency care were mentioned. The midwives said that staff who do not adhere to the unit’s way of working, because they do not agree with the working method, can actually counteract the care. They also described concerns that collaboration with the staff at the neonatal intensive care unit could pose a problem. Further, they said that, at a resuscitation table, all the equipment is in place, but in the birth room, the equipment may be difficult to access or may not even be present in the room, which was described by some of the midwives in unit A. One midwife said that there was a risk of the baby falling to the floor in connection with the emergency care:

*‘[Neonatal staff] only see disadvantages in the form of this being “new” while we as midwives, we can do something constructive when we're not comfortable with a situation. More education and better collaboration are needed to make it work.’*

Introducing a new working method and new routines for emergency care entails a major reorganization in which it becomes important to collaborate with, and allocate work to, team members. Moreover, functioning technical equipment is a prerequisite, and so is the training of staff.

### Benefits of reduced separation

Many of the midwives believed that there are no disadvantages, for the newborn baby, in performing emergency care on the mother’s breast or on a resuscitation table inside the birth room. When a baby is resuscitated in the birth room, it is possible for it to remain on SSC with the mother, according to several of the midwives. This gives the baby security, a connection, and warmth, and leads to improved breathing, faster recovery, good blood glucose levels, and a calmer baby. However, the midwives had differing opinions about what is of benefit to the baby. One midwife said that the benefits of a baby receiving resuscitation at the mother’s bedside were not clear, while another believed that some SSC can be maintained even outside the birth room if one parent is standing next to the baby. The fact that the baby can hear its parents’ familiar voices, which increases its feeling of safety, was also considered positive. Late cord clamping and sustained placental transfusion were seen as an advantage for the baby, as they would result in increased blood flow and a higher hemoglobin value in the long run. Some midwives answered the question about late cord clamping, saying that it gives better oxygen saturation and a faster recovery. One midwife also highlighted the benefit of late cord clamping to preterm babies, saying that it increases their survival rate.

Most midwives considered it positive for the mother if resuscitation of the baby took place inside the birth room. It allowed the mother to feel more present with what was happening to her baby and what measures were being taken to help it. Obtaining direct firsthand information about the baby in this way, and thus avoiding uncertainty, was considered to increase security for both the mother and her partner. Participation in care and the increased security of not having to be separated from the baby were also described by many to improve the connection between the mother and her child, and to facilitate breastfeeding. Being present when the baby recovers, and then being physically close, was likewise considered positive:

*‘Not to be separated from their baby, to be able to see what is being done and what is happening … get the opportunity to be involved when the baby picks up and utters its first cry.’*

Many midwives thought that taking care of the baby in the birth room, and thus avoiding separation, reduces the traumatic experience that the baby is unwell and needs help to survive. The separation that occurs when the baby is transferred from the birth room was considered by some midwives to be more traumatizing than seeing what happens. Unrealistic notions about emergency care may then result. Further, when the partner follows the baby out of the birth room, this can, according to some midwives, reduce the feelings of security of the mother who remains behind. When both parents are present in the birth room, they have the opportunity to process the event together post-intervention and may not feel the need to question the procedure at a later point.

## DISCUSSION

This study is, to the best of our knowledge, the first to illuminate midwives’ experiences of, and perception about, mother-baby separation during resuscitation of the baby following birth. The experience of separating the mother–baby dyad, by taking the newborn baby out of the birth room when emergency care is needed, was described by most of the included midwives. However, it was clear that in unit A where resuscitation was performed outside the birth room, midwives saw fewer benefits for the mother and baby. In organizations where separation is the norm, either because of cultural norms or for physical reasons (e.g. lack of space in the birth room), midwives might continue to practice cessation of SSC and separate mother and baby as motherside resuscitation is considered more difficult to perform compared to resuscitation in a separate room. This has also been highlighted in a cross-sectional descriptive study where some midwives described disadvantages of motherside resuscitation in hospital settings, primarily related to ergonomics and the resuscitation setup limiting adequate access to the infant with the cord intact^11^.

The impact of the physical features of the birth room on the care provided following birth was described in the present study. For instance, the midwives mentioned that lack of space can lead to a suboptimal working position and can thus make it difficult to keep the baby in a good position when ventilation is performed while on SSC. Some felt that lack of space entailed challenges that made it difficult for staff to perform the emergency care in the birth room. The midwives also described the risk of forgetting the mother’s care needs. They emphasized the importance of respect for and responsiveness to the parents’ questions and requests, partly for the integrity of the mother, but also so that the family’s wishes regarding the care intervention were met. This was seen as difficult and as requiring experience and training for it to be performed properly^13,17^.

It has previously been reported that separation interferes with optimal care for the newborn baby^2^. Despite the consequences of separating mother and baby, the midwives in the present study demonstrated a difference in regard to the care culture, and mother–baby separation, between the two included units. The size of the rooms was equal in units A and B, but routines and working methods differed. Some of this difference was due to how the units were differently designed, mainly regarding where the resuscitation table was located. In unit A, the organization of newborn emergency care was not in accordance with best evidence, and as a result, staff at this unit continued to separate the mother–baby dyad. A recent study reports that motherside resuscitation is possible when wanting to make care as optimal as possible for the newborn baby and the mother^18^. To separate the baby from its mother must never jeopardize medical safety. Before motherside resuscitation is possible, the rooms must be rebuilt to make this possible. To not separate is of huge importance, but the care must be medically safe, both for the baby and the mother, and all necessary equipment inside the delivery room must be there so that resuscitation can be possible, without delays. This would be best practice.

Many factors influence midwives’ decisions when a newborn baby is critically ill following birth, experience and intuition are important factors in this decision-making process, and, if clinical practice and guidelines are changed, the decision-making will be considerably affected^19^. It is well known that it can be difficult to change practice in well-established organizations and that the success of implementation of new working methods is dependent on behavior change among the healthcare personnel, such as the midwives in our study^20^. Therefore, translating research evidence into practice is not easy, but the midwives who work with the mothers and babies have a central role in this process. Winston Churchill once famously said, ‘We shape our buildings, and afterwards our buildings shape us’. Perhaps it is the same with emergency care of a baby after birth: if we build new birth units where all resuscitation can be performed motherside, this will change the midwives’ working methodology and their knowledge about, and attitudes to, this procedure. A new working methodology and new work routines also present challenges for the healthcare personnel. As Porter and Macintyre^21^ remark, women tend to assume that whatever system of care is provided has been well thought out and is therefore likely to be the best one and where they express a preference, it is generally for whatever arrangements they have experienced rather than for other possible arrangements.

Further, change of practices need to be supported by all members of the multidisciplinary team (hospital managers, seniors obstetric/pediatric consultants, midwifery management neonatal team etc.), not just the midwives. This will require education and support from, and for all, stakeholders.

The practice of separating mother and baby has been the custom for many decades, since the introduction of incubator care and the use of infant formula^2^. However, contemporary childbirth/neonatal care should be family-centered as well as evidence-based. To justify separation at birth by highlighting the need for resuscitation should no longer be accepted. A growing body of evidence reports that intact cord resuscitation supports the process of physiologic neonatal transition and is beneficial for non-vigorous newborn infants^22,23^. If motherside resuscitation cannot be practiced for maternal reasons, specially designed equipment or trolleys can facilitate resuscitation, in the same room, with the placental and cord circulation intact^18^.

It was also described in the present study that the midwives who had experience of taking compromised babies to the emergency room, sometimes awaited urgent decisions and actions when they felt that the baby was recovering. Fulton et al.^13^ discuss whether the midwifery perspective, that separation should only be performed if there is compelling evidence, means that midwives prioritize evidence differently from other care providers when considering policies and procedures. In line with the opinion of several of the interviewed midwives, it has been emphasized elsewhere that the midwife should strive for zero separation and promote SSC after birth wherever possible^2^. The results of the present study show that unit B managed to achieve zero separation to a greater degree than did unit A, partly because there is no resuscitation table in the birth rooms in unit A, and mother and baby may therefore need to be separated to perform emergency care of the baby.

The midwives in both units agreed that separation of mother and baby after birth should be kept to a minimum, which is reinforced by research that shows that SSC and, therefore, no separation, is an important part of the newborn’s transition to extrauterine life^3,24^. When a mother is separated from her newborn baby, she may experience being deprived of the first minutes with the baby, which can make bonding more difficult^7^. Separation can be traumatic for the mother just as for the baby, since it involves an imposed stress, which in turn can affect the baby’s brain development^2,25^. The midwives expressed that if the parents were present at the resuscitation table this could enhance bonding; however, it was also suggested that parents when witnessing resuscitation of their newborn may experience it as traumatic. Previous studies have addressed this and report that parents are generally positive about witnessing resuscitation. Knowing what is happening and seeing the staff doing their best can comfort them. Also being able to touch and see their baby can make them feel involved, as a family, in the first moments of their baby’s life^11^. However, further investigation of mothers’ and parents’ perceptions of what they think is beneficial for babies/mothers/parents in regard to mother–baby separation during resuscitation of the baby following birth, is warranted.

### Strengths and limitations

In this study, we invited all midwives working in units A and B to participate, aiming to include all midwifery experiences, both positive and negative. This strategy has given breadth to the findings, which is a strength of the study. On the other hand, the study did not include, for example, nurses from the neonatal ward. Including them might have given a wider perspective on the work towards reducing separation between mother and baby after birth, as nurses are a staff group involved in neonatal emergency care that could be started both inside and outside the birth room.

The questionnaires were sent to 81 midwives in unit A and unit B and the response rate was lower in unit B than in unit A. The reason for the lower response rate in unit B is unknown as the midwives did not have to give a reason for not wanting to participate. The assurance that they would participate anonymously gave the participants a greater sense of security when responding to questions and therefore possibly resulted in more honest answers, as responses could not be traced back to a specific midwife. The answers tended to be brief and curt, which made the analysis challenging. Short sentences and single words can mean lost information, which can lead to misinterpretation. An interview method would have provided the opportunity for follow-up questions, possibly leading to more detailed answers. The study is based on both quantitative and qualitative data, an approach that contributes to a wide range of findings^14^.

### Clinical implications

Continuous education and training are needed to respond to midwives’ concerns and uncertainty about new working methods and routines. If this education and training is not provided, anxiety may prevent the midwives from carrying out emergency care in the birth room, which may mean that they instead choose to transfer the baby to the emergency room and thus separate mother and baby. Further, this education and training needs to include all team members who care for mother and baby at birth (medical team, neonatal team, etc.), not just midwives. Different conditions prevail in the neonatal units in Sweden regarding resuscitating a newborn inside the birth room, including the possibility of having video surveillance as well as space for resuscitation tables in some, but not all, of the birth units. Therefore, the care is not equal. To achieve equality, all birth units should be evidence-based. They should aim to reduce the separation between mother and baby after birth as far as possible. To broaden knowledge about the cooperation with other health professionals in the birth room, further research may be needed.

## CONCLUSIONS

There are good opportunities to reduce separation of mother and baby after birth; training, knowledge, education, and the right environmental conditions are important factors in successfully implementing new ways of working. In a birth unit where separation is the norm, midwives might continue to practice cessation of SSC and separate mother and baby, as motherside resuscitation is considered more difficult to perform than resuscitation in a separate room. It is important that birth units carefully think through and plan for their approach to neonatal emergency care, including separation or not of the mother–baby dyad.

## Data Availability

The data supporting this research are available from the authors on reasonable request.
